# Thermodynamics of emergent magnetic charge screening in artificial spin ice

**DOI:** 10.1038/ncomms12635

**Published:** 2016-09-01

**Authors:** Alan Farhan, Andreas Scholl, Charlotte F. Petersen, Luca Anghinolfi, Clemens Wuth, Scott Dhuey, Rajesh V. Chopdekar, Paula Mellado, Mikko J. Alava, Sebastiaan van Dijken

**Affiliations:** 1 Advanced Light Source, Lawrence Berkeley National Laboratory (LBNL), 1 Cyclotron Road, Berkeley, California 94720, USA; 2 NanoSpin, Department of Applied Physics, Aalto University School of Science, P.O. Box 15100, FI-00076 Aalto, Finland; 3 COMP Centre of Excellence, Department of Applied Physics, Aalto University, P.O. Box 11100, FI-00076 Aalto, Espoo, Finland; 4 Dipartimento di Fisica, Università di Genova, via Dodecaneso 33, I-16146 Genova, Italy; 5 Department of Emerging Materials Science, Daegu Gyeongbuk Institute of Science and Technology (DGIST), Daegu 711-873, Korea; 6 Molecular Foundry, Lawrence Berkeley National Laboratory (LBNL), 1 Cyclotron Road, Berkeley, California 94720, USA; 7 Department of Materials Science and Engineering, University of California, Davis, California 95616, USA; 8 School of Engineering and Sciences, Adolfo Ibáñez University, Diagonal Las Torres, 2640 Peñalolén, Santiago, Chile

## Abstract

Electric charge screening is a fundamental principle governing the behaviour in a variety of systems in nature. Through reconfiguration of the local environment, the Coulomb attraction between electric charges is decreased, leading, for example, to the creation of polaron states in solids or hydration shells around proteins in water. Here, we directly visualize the real-time creation and decay of screened magnetic charge configurations in a two-dimensional artificial spin ice system, the dipolar dice lattice. By comparing the temperature dependent occurrence of screened and unscreened emergent magnetic charge defects, we determine that screened magnetic charges are indeed a result of local energy reduction and appear as a transient minimum energy state before the system relaxes towards the predicted ground state. These results highlight the important role of emergent magnetic charges in artificial spin ice, giving rise to screened charge excitations and the emergence of exotic low-temperature configurations.

## Abstract (web summary)

Inspired by the physics of bulk frustrated materials, arrays of coupled nanomagnets have been widely explored for the study of collective ordering and emergent behaviour. Here, the authors demonstrate interaction-driven charge screening in a thermally active artificial spin ice lattice.

## Graphical abstract

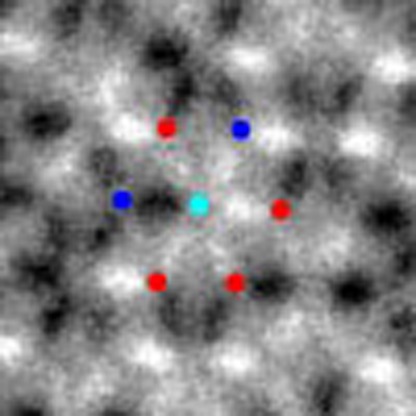

Frustration stands for the inability of entities within a given system to simultaneously minimize their interactions, thus hindering the system to reach its lowest energy state^1^,^2^,^3^. In frustrated magnetism, the formation of simple ferro- or anti-ferromagnetic configurations is prevented, which leads to the emergence of new phases that cannot be described in a conventional manner, such as the infamous spin ice phase in magnetic pyrochlore materials^1^, which was successfully described using the concept of emergent magnetic charges^4^. The introduction of artificial spin systems has provided the prospect to investigate frustration directly with real-space imaging techniques^5^,^6^,^7^,^8^,^9^. Artificial spin ices consist of lithographically patterned nanomagnets arranged in two-dimensional geometries, such as the artificial square ice^6^,^8^,^10^,^11^,^12^ and artificial kagome spin ice^5^,^9^,^13^,^14^. The most recent emergence of artificial spin ices with thermally activated moment fluctuations^5^,^6^,^9^,^10^,^12^ not only delivered evidence on the important role of emergent magnetic charge defects in thermal relaxation processes^6^,^10^, but also that screening effects between emergent magnetic charges are crucial for charge-ordering phenomena in a two-dimensional shakti lattice^15^ and the possible emergence of polaronic states in artificial spin ice^16^. This raises the question, whether these emergent magnetic charge screening phenomena are just a result of the lattice geometry or originate from a minimization of interaction energies between the involved entities.

Here we provide direct visual evidence of the real-time formation and annihilation of screened states, which appear as a result of local energy minimization of interactions between the entities within a new two-dimensional artificial frustrated system consisting of nanomagnets occupying the sites of a dice lattice, thus referred to as the dipolar dice lattice

## Results

### The dipolar dice lattice

**Figure 1:**
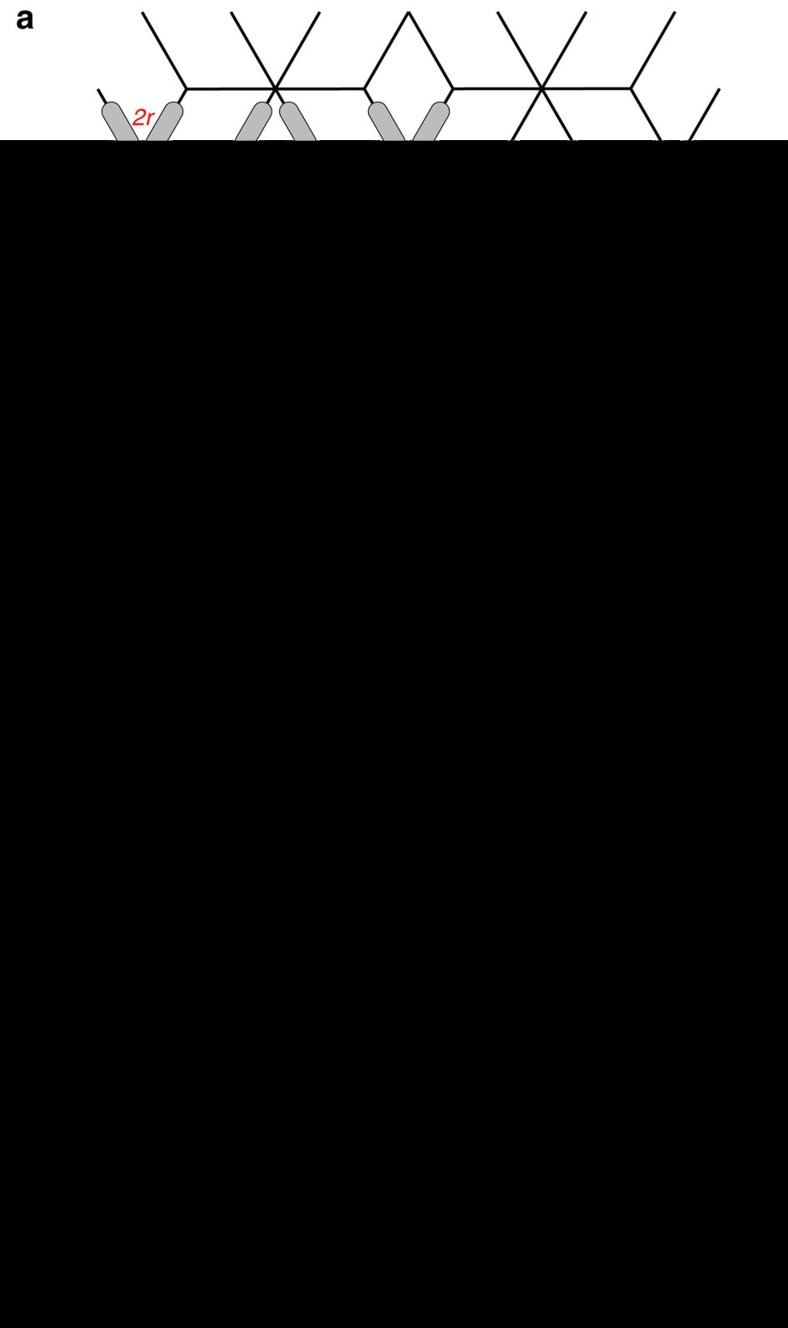
The dipolar dice lattice. (**a**) Schematic of a two-dimensional dipolar dice lattice with elongated nanomagnets. Being a system with mixed coordination numbers, the lattice exhibits z6 vertices, where six nanomagnets meet, and z3 vertices where three nanomagnets meet. While the background lattice is defined by a lattice constant *a*=500 nm, the dipolar coupling between the nanomagnets is varied by changing the lattice spacing (2*r*=500, 545 and 600 nm). (**b**) Vertex types (at z6 vertices) listed with increasing energy from V1 to V8 together with their respective degeneracies *g* and their net emergent magnetic charges *Q*_*z*6_. In a low-energy configuration, z3-vertices obey the ice rule (two-in-one-out or *vice versa*) with a net magnetic charge *Q*_*z*3_=±*q*. (**c**) X-ray magnetic circular dichroism (XMCD) images of thermally activated dipolar dice lattice. Nanomagnets with moments pointing towards the incoming X-rays (indicated by red arrow) appear dark, while moments opposing the incoming X-rays appear bright. An emergent magnetic charge defect at a z6 vertex site with an overall charge *Q*_*z*6_=±2*q* (cyan blue circle) is initially unscreened, as it is surrounded by three +*q* and three −*q* charges at the z3 vertices (at *t*=0 s). Eventually, a +*q* at a z3 vertex is transformed into a −*q* charge, which results in a screened state (at *t=*7 s) with an overall charge of 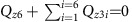. The screened charge defect decays at *t*=28 s. The yellow scale bar indicates a length of 545 nm.

Being a lattice with mixed coordination numbers, the dipolar dice lattice (Fig. 1a and Supplementary Fig. 1) exhibits two types of vertices, namely z3 vertices, where three nanomagnets meet (green circles in Fig. 1a) and z6 vertices, where six nanomagnets meet (pink circles in Fig. 1a). Energetically, eight different vertex types with increasing energy from V1 to V8 can be identified for z6 vertices (Fig. 1b), which is similar to the categorization of triangular artificial spin ice energy configurations^17^. At the z3 vertices, moment configurations that minimize the dipolar energy obey the so-called ice rule of either two moments pointing into the vertex and one out or *vice versa*^5^,^18^ (Fig. 1b). In a magnetic charge representation, where each magnetic moment is replaced by a pair of magnetic charges ±*q* residing at the ends of every nanomagnet^4^,^13^, each z3 vertex obeying the ice rule has a net negative or positive magnetic charge *Q*_*z*3_=±*q*. Excitations out of this spin ice manifold will violate the ice rule and exhibit *Q*_*z*3_=±3*q*. Similarly, at the z6 vertices, V1, V2 and V5 vertex types exhibit no emergent magnetic charge, while V3, V4 and V6 have a magnetic charge of *Q*_*z*6_=±2*q*. Emergent magnetic charge defects at z6 vertex sites, which can be seen as local impurities, get eventually screened by environmental z3 charges.

### Low-temperature configurations

**Figure 2:**
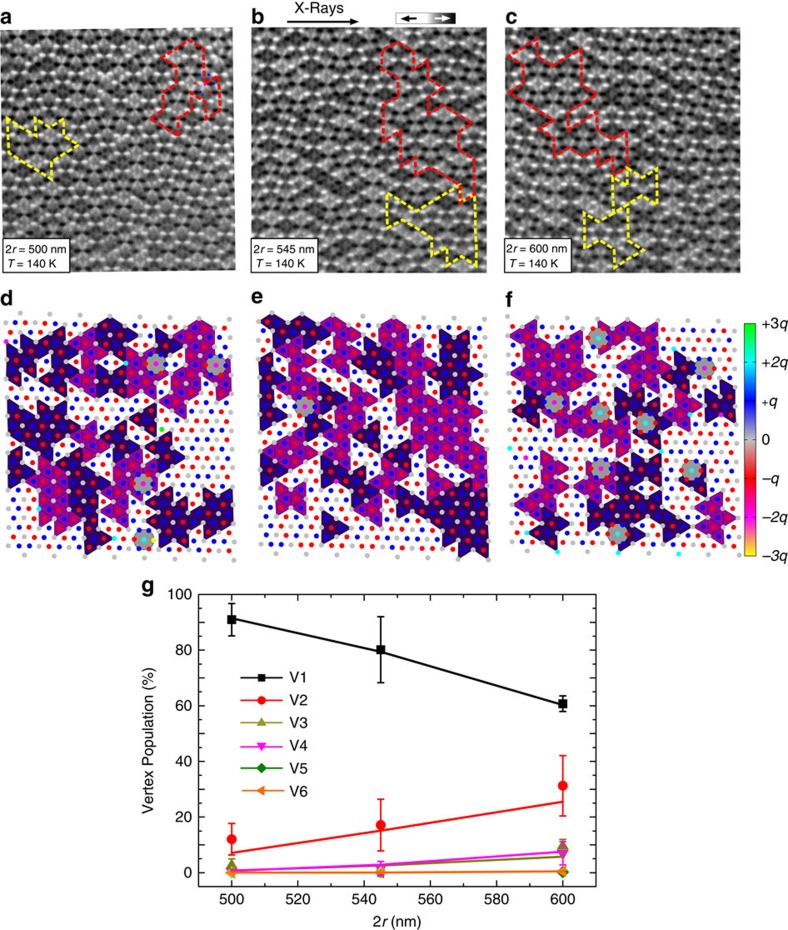
Equilibrium configurations of the dipolar dice lattice. (**a**–**c**) XMCD images (field of view=12 μm) of frozen-in configurations (*T*=140 K) of dipolar dice lattices with various lattice spacings (2*r*=500, 545 and 600 nm). Nanomagnets with moments pointing towards the X-rays appear dark, while moments opposing the incoming X-rays appear bright. (**d**–**f**) Vertex charge representation of the XMCD images. The results reveal a tendency of the system to exhibit V1 vertices at z6 sites with zero net magnetic charge (small grey dots). In addition, the ±*q* charges residing at z3 vertices tend to form charge-ordered domains (highlighted with deep violet- and and deep purple triangles). An increase of the lattice spacing leads to more ±2*q* charge defects emerging at z6 vertex sites (cyan or magenta dots). The majority of charge defects are screened by a cloud of ±*q* magnetic charges at z3 vertices (red and blue dots). (**g**) Average experimental vertex-type populations at z6 sites plotted as a function of lattice spacing 2*r* (symbols), showing good agreement with equilibrium populations obtained from Monte Carlo simulations (lines). The error bars represent standard deviations originating from four experimental observations.

As a first step, we investigate the low-temperature equilibrium states of dipolar dice lattices with 2*r*=500 nm, 545 nm and 600 nm. We focus on arrays consisting of nanomagnets with length *L*=300 nm, width *W*=100 nm and thickness *d*=2.5 nm (see Methods). The blocking temperature *T*_B_, which we define as the temperature at which moment re-orientations start to occur within the timescale of a single photoemission electron microscopy (PEEM) measurement (7–10 s per images)^6^,^19^, is determined to be 160 K. Analysing the low-temperature configurations achieved after cooling the sample from 350 to 140 K (Fig. 2a–f), we find for all lattice spacings that the zero-charge V1 vertex types dominate at z6 vertices (Fig. 2g), while z3 vertices show a clear tendency towards obeying the ice rule, which is manifested by values close to 0.333 for the *C*_*αγ*_ nearest-neighbor correlation function (see Methods and Supplementary Fig. 2). The ±*q* charges residing at z3 vertices (blue and red dots in Figs 2d–f) exhibit a staggered charge-ordering, where each +*q* charge is surrounded by three neighbouring −*q* charges or *vice versa*. Similar to the so-called spin ice II phase in artificial kagome spin ice^20^, there are two degenerate configurations for this charge-ordered ground state (Supplementary Fig. 3). The two configurations lead to the formation of domains, as illustrated by the deep violet- and purple colours in Fig. 2d–f. At the edges of the charge-ordered domains, we encounter magnetic charge defects with ±2*q* charges at z6 vertex sites (cyan and magenta dots in Figs 2d–f and Supplementary Fig. 4). A majority of these charge defects (55–65%) appear to be fully screened by their surrounding z3 charges (highlighted with large grey circles in Fig. 2d–f). When plotting the vertex populations as a function of lattice spacing 2*r* (Fig. 2g), we find that the experimental observations are in good agreement with Monte Carlo simulations based on a model in which each nanomagnet is replaced by an infinitesimally thin dipolar needle^20^,^21^ (see Methods).

### Thermal relaxation in the dipolar dice lattice

**Figure 3:**
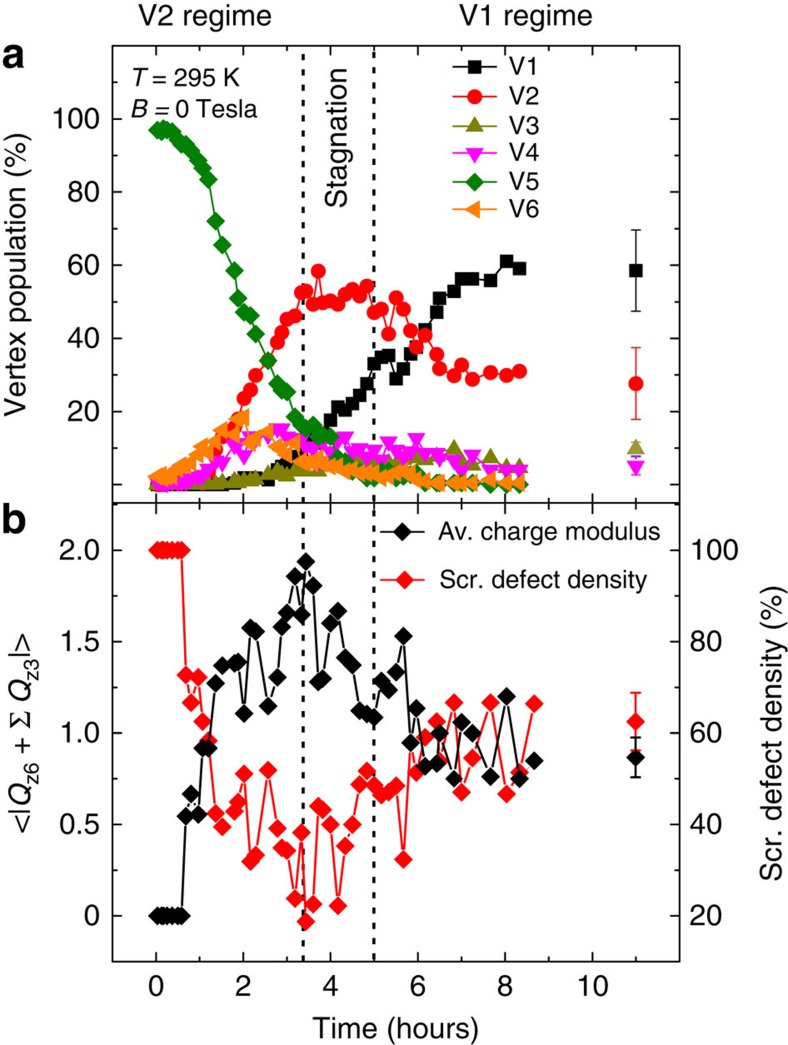
Thermal relaxation of the dipolar dice lattice. (**a**) Temporal evolution of vertex-type population at z6 sites at *T*=295 K. Starting from a fully saturated state (100% V5 vertex population), the system relaxes via thermally-driven moment re-orientations in the patterned nanomagnets. The relaxation process involves three distinct regimes: the V2-regime marked by a strong initial increase in V2 vertices, followed by the stagnation regime in which the V2-vertex population remains constant, and, finally, the V1-regime where the population of V1 vertices rises at the expense of all other vertex types. The system approaches its low-energy equilibrium configuration in the V1 regime after 11 h. (**b**) Average charge modulus 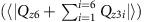 plotted as a function of time (black diamonds) together with the corresponding density of screened magnetic charges (red diamonds). In equilibrium, by recording image sequences, consisting of ten images per sequence, we determine the average equilibrium vertex populations, charge modulus and screened defect density with respective standard deviations given as error bars.

Focusing on a lattice with 2*r*=545 nm and a film thickness of *d*=2.8 nm, we visualize how a thermally active dipolar dice lattice responds after being perturbed by an external magnetic field. In the experiment, the system is kept at a constant temperature of *T*=295 K (*T*_B_=285 K) and a magnetic field of 30 mT is shortly applied along the incoming X-ray direction by an external magnet. After the field is switched off, the system thermally relaxes towards its equilibrium configuration (Supplementary Movies 1 and 2). In Fig. 3, we plot the z6-vertex type population as a function of time. Starting from a 100% V5 vertex background, we find that the initial relaxation phase is marked by an increase in the V2 vertex population. We refer to this initial phase (the first 3.3 h after the magnetic field pulse) as the V2 regime, which also includes a rise and drop in charged V4 and V6 vertices (*Q*_*z*6_=±2*q*). The V2-regime is followed by a stagnation regime where the V2, V4 and V6 populations remain constant, while the population of lowest-energy V1 vertices increases. This change in vertex populations can be understood by the fact that a V2 transforms into a V1 or *vice versa* via the creation and annihilation of emergent magnetic charge defects (V3, V4 and V6). Following the stagnation regime, the V1 vertex population continues to rise while all other vertex populations decrease until the system reaches its equilibrium state (V1 regime). Kinetic Monte Carlo simulations of the relaxation curves are shown in Supplementary Fig. 5.

### Real-time dynamics of emergent magnetic charge screening

Quantitatively, magnetic charge screening can be characterized by calculating the average modulus of z6 charge defects with *Q*_*z*6_=±2*q* and the six z3 vertex charges surrounding them, 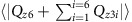 (Fig. 3b). At the early stages of the relaxation process, an average of zero is obtained (100% screening), which is due to charge defects arising from the initial charge-ordered saturated state. As the system relaxes, the magnetic charge defects at z6 vertices emerge out of a non charge-ordered state, and are not immediately screened. Since the number of defects is increasing (and it takes some time for a defect to become compensated), the density of screened charge defects (with respect to all ±2*q* defects) decreases during this phase. Correspondingly, the average charge modulus increases, reaching a maximum at the end of the V2 regime. After this, an increasing number of emerging charge defects at z6 vertices get screened by environmental z3 charges until an equilibrium state with a screening percentage of about 60% is reached. This percentage of screened charge defects is substantially higher than 23%, which is calculated for the case of pure statistical switching in the dipolar dice lattice (Supplementary Fig. 6). This result evidences the role of emergent magnetic charges as a relevant degree of freedom, so that when a non-zero charge defect emerges, it has a tendency to become screened by environmental magnetic charges, thus forming a polaronic state^16^. Eventually, most of the ±2*q* charges disappear.

### Thermal stability of screened charge defects

**Figure 4:**
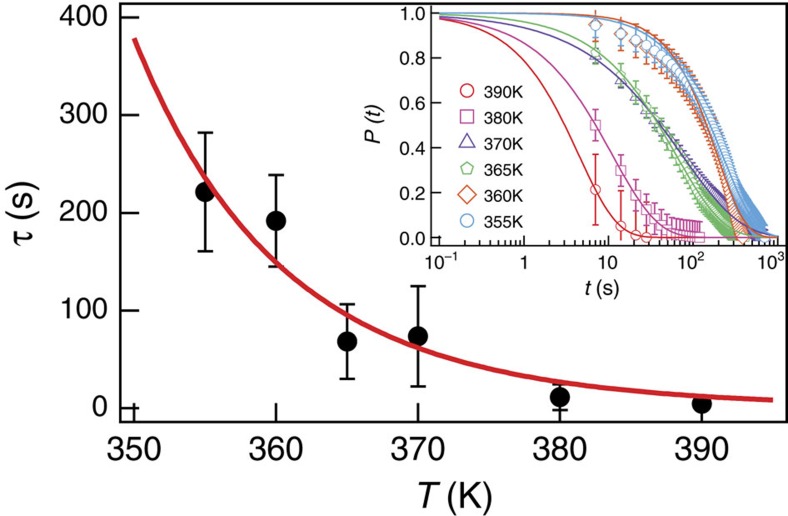
Thermal stability of screened magnetic charges. Experimental lifetime of screened magnetic charge defects arising at z6 vertex sites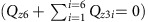 plotted as a function of temperature (black dots) together with an Arrhenius-type fit. The characteristic lifetime at each temperature is extracted by fitting the probability of screened charge defects using 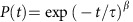 (inset). Error bars are estimated by employing a bootstrapping algorithm with a confidence interval of 95% (see Methods).

Further quantitative insight into the nature of the magnetization dynamics involving screened and unscreened magnetic charge defects is gained by taking a closer look into the temperature dependence of their thermal stability in equilibrium. Focusing on a lattice spacing of 2*r*=545 nm and a film thickness of *d*=3 nm (*T*_B_=355 K), we imaged thermal fluctuations at various temperatures between 355 and 390 K. From each of the recorded data sets, the probability *P*(*t*) of observing screened (or unscreened) magnetic charge defects as a function of time is extracted (see inset in Fig. 4). For each individual probability distribution, the error is assessed by calculating a confidence interval of 95% (ref. 22) (Supplementary Information). The obtained probabilities are fitted using a Kohlrausch function^23^ 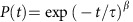, with β being the stretch parameter^2^,^23^,^24^ (Supplementary Fig. 7) and *τ* the characteristic lifetime of the observed state. All fitting curves lie well within the experimental error bars. The characteristic lifetimes *τ*(*T*) are plotted as a function of temperature in the main panel of Fig. 4. Here, the error is assessed by employing a bootstrapping algorithm with a confidence interval of 95% (ref. 22) (Supplementary Information). The curve is then fitted by an Arrhenius-type function 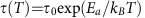. Here, *τ*_0_ represents an attempt time that depends on the size, shape and material of the nanomagnet^25^. Since decay of screened and unscreened magnetic charge defects involves thermally-driven magnetic switching in a lattice of identical permalloy nanomagnets, we assume it to be the same for both states. Using an attempt time of *τ*_0_=10^−12^ s (refs 5, 6), we obtain an average activation energy of 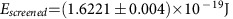 for screened charge defects, a value that corresponds closely to the energy barrier for magnetic switching in an individual nanomagnet. Similarly, we are able to obtain the average activation energy for unscreened defects, 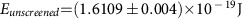. These results show that screened charge defects are thermally more stable. The energy difference, 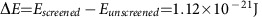, represents an average lowering of the dipolar interaction energy when a polaron-like magnetic excitation is formed in the spin ice lattice. We note that the derived value of Δ*E* does not vary sensitively with the selected attempt time (Supplementary Fig. 8).

## Discussion

Our experimental findings provide first real-time visual evidence that the formation of screened magnetic charge excitations in artificial spin ice emerge as a consequence of local energy minimization and not just as a result of lattice geometry. We anticipate our work to stimulate further studies on the role of screening phenomena in the ordering of spin ice systems with mixed coordination numbers^15^,^16^ and the exploration of the dynamic response of screened charge defects with potential for applications in reprogrammable magnonics^26^,^27^.

## Methods

### Sample fabrication

The artificial spin ice samples were fabricated using an e-beam lithography process similar to previous work^5^,^6^,^19^: A silicon (100) substrate was first spin-coated with a 70-nm-thick layer of polymethylmethacrylate resist. The dipolar dice lattices were then defined onto the sample with a VISTEC VB300 electron beam writer. Next, a ferromagnetic permalloy (Ni_80_Fe_20_) film was thermally evaporated at a base pressure of 2 × 10^−7^ torr, which was followed by lift-off in acetone at a temperature of 50 °C. Thermally-driven moment fluctuations in the artificial spin ice samples were realized by fabrication of ultrathin nanomagnets with length *L*=300 nm and width *W*=100 nm. The thickness of the patterned nanomagnets discussed in this work ranged from 2.5 to 3 nm. The corresponding blocking temperatures varied from 160 to 355 K.

### X-ray photoemission electron microscopy

The experiments were performed at the PEEM3 beamline at the Advanced Light Source (ALS)^28^. Magnetic imaging was performed taking advantage of X-ray magnetic circular dichroism (XMCD) at the Fe L3-edge^29^. The resulting contrast is a measure of the projection of the magnetization on the X-ray polarization vector, so that nanomagnets with a magnetization parallel or antiparallel to the X-ray polarization either appear black or white. Nanomagnets with an angle of ±60° or ±120° with respect to the incoming X-rays appear dark- or light grey, respectively.

### Magnetic moment- and charge correlations

In addition to characterizing the moment configurations achieved with vertex type statistics (Fig. 2g), an elegant way to extract quantitative information is to calculate magnetic moment- and charge correlations (Supplementary Fig. 2). This correlation measure is calculated using the same method as in previous work^5^,^7^,^9^,^15^. Two moments with a positive inner product (*m*_i_·*m*_j_>0) are given a correlation value of *C*_ij_=+1, and a correlation value of *C*_ij_=−1, if the inner product is negative. Similarly, the charge–charge correlations are calculated. Charge pairs with opposite signs are given a correlation value of +1 and charge pairs with the same sign are given a correlation value of −1. An average is then calculated for the whole moment- and charge configuration achieved in the experiments. The number of moments used in the statistics is ∼1,100 per image.

### Bootstrap method

For the Arrhenius plot, the error is estimated by employing a bootstrapping algorithm with a confidence interval of 95% (ref. 22). At each temperature, one thousand calculated data sets are generated randomly from the existing experimental data. These calculated data sets are then fitted individually to a stretched exponential 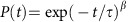, in order to obtain characteristic lifetimes. With this, at a specific temperature *T*=const, a distribution of one thousand lifetimes 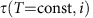 is obtained, where the identifier *i* denotes an integer in the range of [1, 1000] referring to a specific calculated set of data. The distribution is normalized and fitted to the Gaussian function, from which the bootstrapped mean value as well as the confidence interval is assessed. The experimental values of *τ* are well in the range of the confidence interval of the bootstrapped mean value, and the Arrhenius fit lies within the error of the experimental values (Fig. 4).

### Simulations

The system was simulated using kinetic Monte Carlo, modelling each nanomagnet as an infinitesimally thin dipolar needle with a length of *L*=300 nm. To take the nanomagnets' spatial extent into account, each nanomagnet is assumed to have uniform magnetic moment density 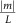. This is equivalent to replacing each nanomagnet by two opposite magnetic charges ±*q* residing at the ends of each nanomagnet^20^,^21^. The dipolar interaction is described by the following Hamiltonian:


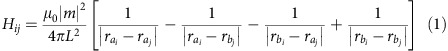


where 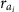 and 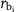 are the locations of the positive and negative magnetic charge on the *i-*th nanomagnet, *μ*_0_ is the magnetic permeability, and 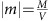 is the magnetic moment of each nanomagnet with *M* being the saturation magnetization and *V* the nanomagnet volume.

In general, the kinetic Monte Carlo method evolves the system through single spin flips, where the probability of a given spin flip move is proportional to its rate^5^. The length of each time step is drawn stochastically from the distribution 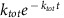, where *k*_*tot*_ is the total rate of a transition from the current state. Assuming the rate of every possible move is known accurately, the simulated dynamics will correctly model the experiment^30^. In the simulations, an Arrhenius-type switching behaviour is assumed for each nanomagnet (*v*=*v*_*0*_ exp (−*E/k*_*B*_*T*)). The reorientation barrier *E* is equal to the intrinsic energy barrier *E*_*0*_ plus half the dipolar energy gain associated with moment reorientations (equation 1). In the kinetic Monte Carlo simulations, *M*=310 kA m^−1^, *E*_0_=0.941 eV, and *v*_*0*_=10^12^ s^−1^ were used. The simulated lattice consisted of 10,800 nanomagnets. Disorder (variation in the intrinsic energy barrier of each nanomagnet) was explored and it was found to have little effect on the temporal evolution of the spin ice system. The results presented in Fig. 2 were calculated by running the kinetic simulations until equilibrium was reached. Kinetic Monte Carlo simulations of relaxation curves are shown in Supplementary Fig. 5.

### Data availability

The data that support the findings in this study are available from the authors on request.

## Additional information

**How to cite this article:** Farhan, A. *et al*. Thermodynamics of emergent magnetic charge screening in artificial spin ice . *Nat. Commun.* 7:12635 doi: 10.1038/ncomms12635 (2016).

## Supplementary Material

Supplementary Information Supplementary Figures 1-8

Supplementary Movie 1 XMCD image sequence of dipolar dice lattice undergoing thermal relaxation from an energetically excited state towards a low-energy state close to equilibrium.

Supplementary Movie 2 Sequence of cropped images of magnetic charge and moment configurations.
