# Supplementary material for: Thermodynamics of emergent magnetic charge screening in artificial spin ice
Source: Nat Commun. 2016 Sep 1;7:12635. doi: 10.1038/ncomms12635 (PMC5040513; doi:10.1038/ncomms12635)
Supplement: Supplementary Information — Supplementary Figures 1-8 [file ncomms12635-s1.pdf]

Supplementary Figures

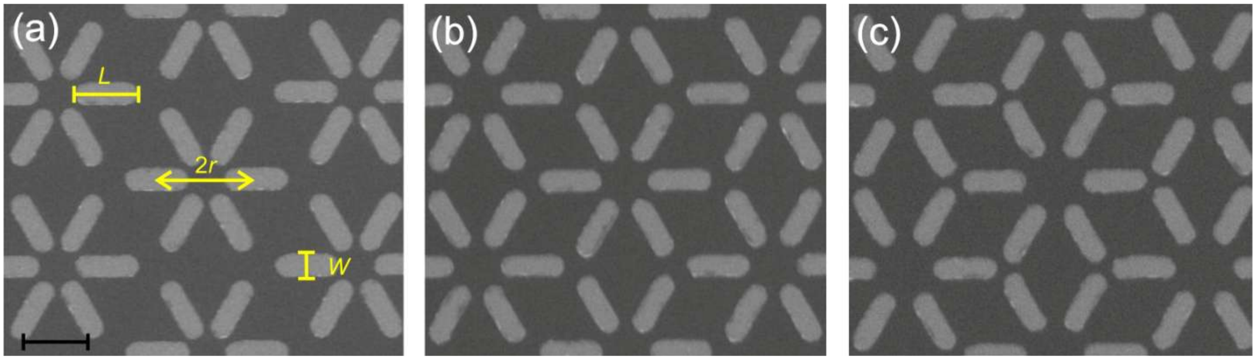

Supplementary Figure 1. The dipolar dice lattice. a-c, SEM images (black scale bar indicates a length of 300 nm) of dipolar dice lattices consisting of nanomagnets with length L = 300 nm and width W = 100 nm. The lattice spacing $2r$ corresponds to 500 nm (a), 545 nm (b) and 600 nm (c).

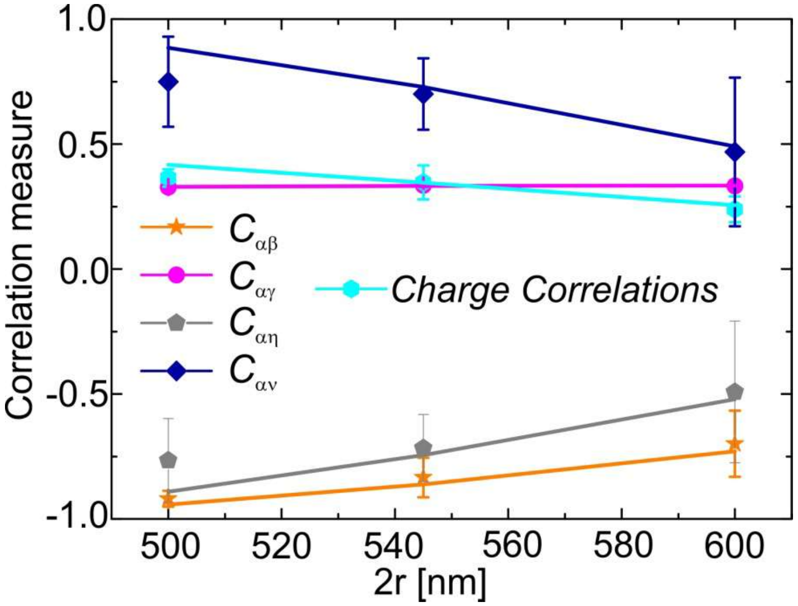

Supplementary Figure 2. Low-energy equilibrium states. Nearest-neighbor moment- and charge correlations obtained from experimental observations (symbols) and simulations (lines). The error bars represent standard deviations originating from four experimental observations.

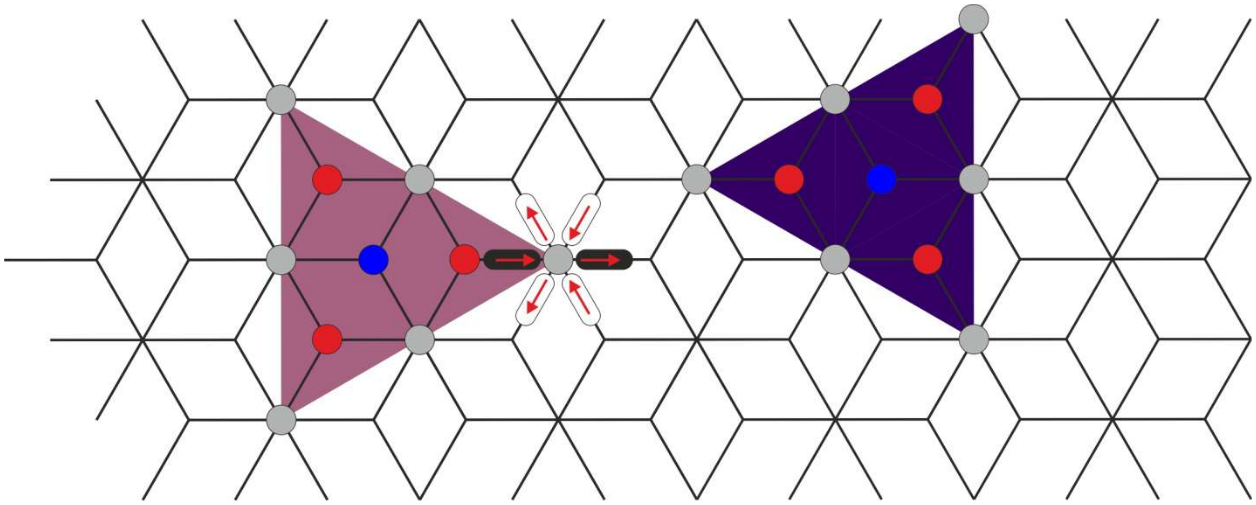

Supplementary Figure 3. Charge-ordered low-energy states in the dipolar dice lattice. In a low energy state, V1 vertices will populate the z6 vertex sites with zero net magnetic charge (grey circles). At the z3 sites, the ice-rule will be obeyed with $\pm q$ charges (blue and red circles) residing at the vertices. These charges will be ordered, so that each positive charge (blue circle) will be surrounded by three negative charges or vice-versa. There are two degenerate ways to achieve this type of charge coordination, either by a right-sided coordination (deep violet triangles) or left-sided coordination (deep purple triangles).

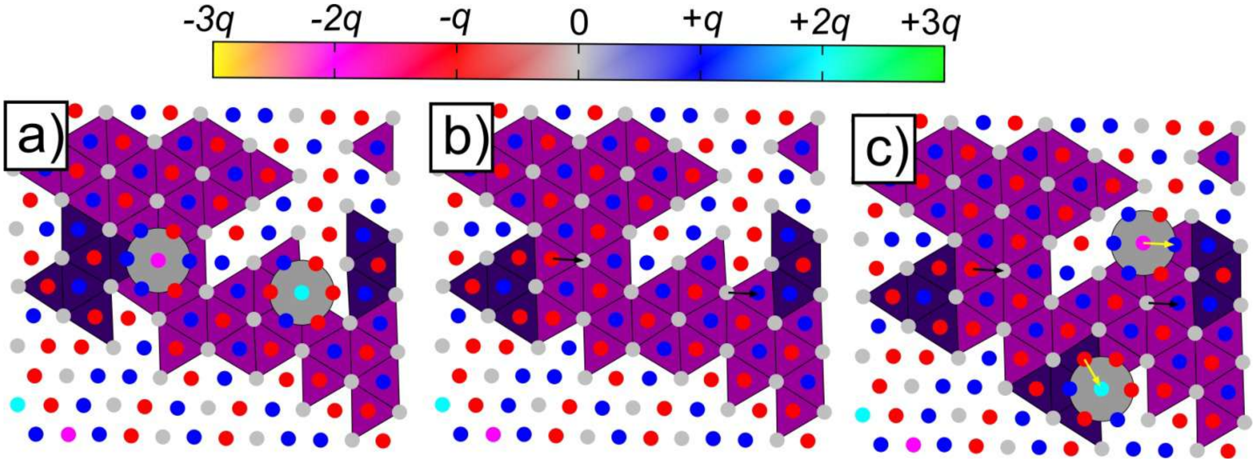

Supplementary Figure 4. Screened charge defects and ground state domain expansion. a, Two screened charge defects reside at the boundary of a larger (charge-ordered) ground state domain (deep violet triangles). **b,** Moment reversals (black arrows) at z6 vertices lead to an expansion of the domain and the annihilation of screened charge defects. **c,** Subsequent moment reversals (white arrows) lead to a further expansion of the charge-ordered domain and the creation and the annihilation of charge defects.

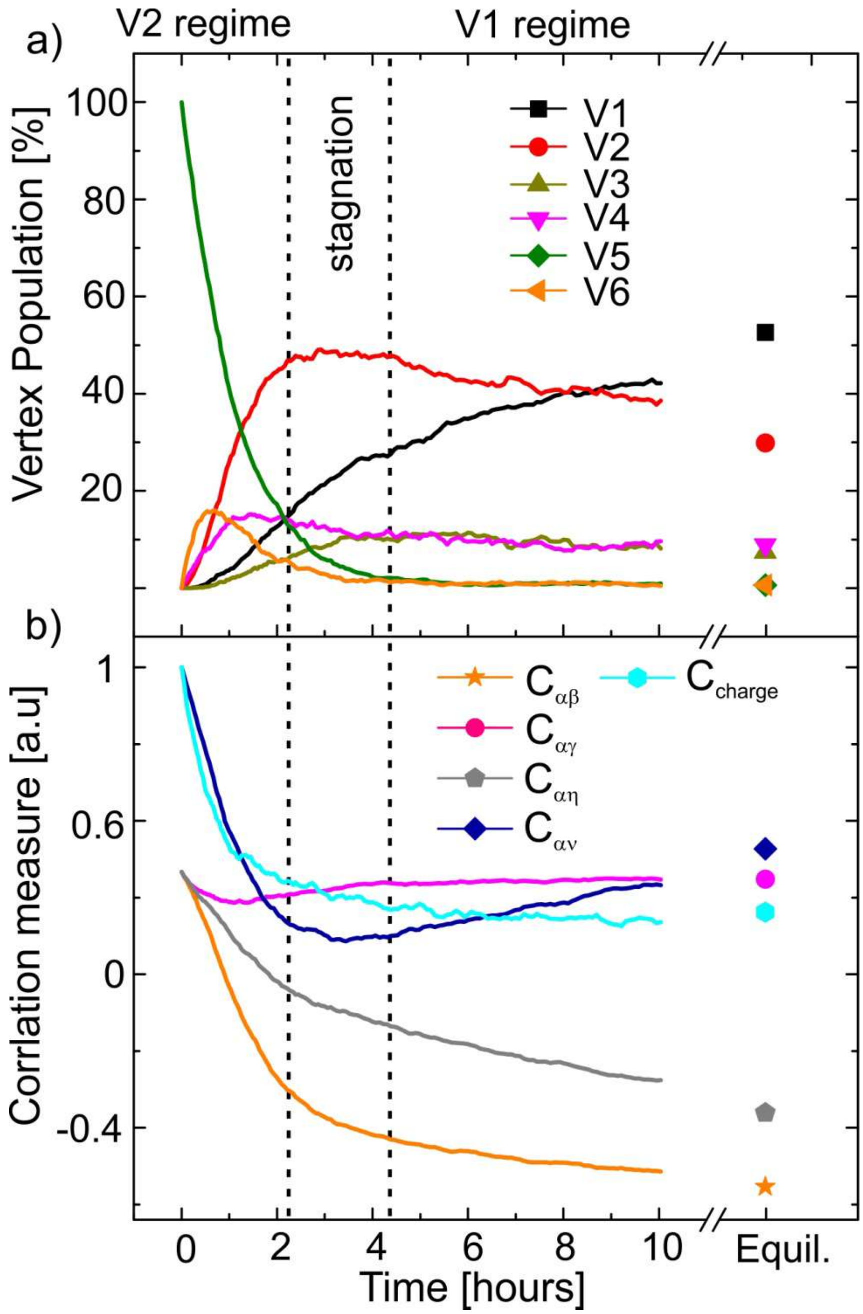

Supplementary Figure 5. Kinetic Monte Carlo simulations of relaxation curves. a, Temporal evolution of vertex type population. **b,** Magnetic charge- and moment correlations plotted as a function of time. Fitting parameters used for kinetic Monte Carlo simulations were M = 310 kAm^{-1}, E_0 = 0.941 eV, $v_0 = 10^{12}$ s^{-1} and T = 295 K.

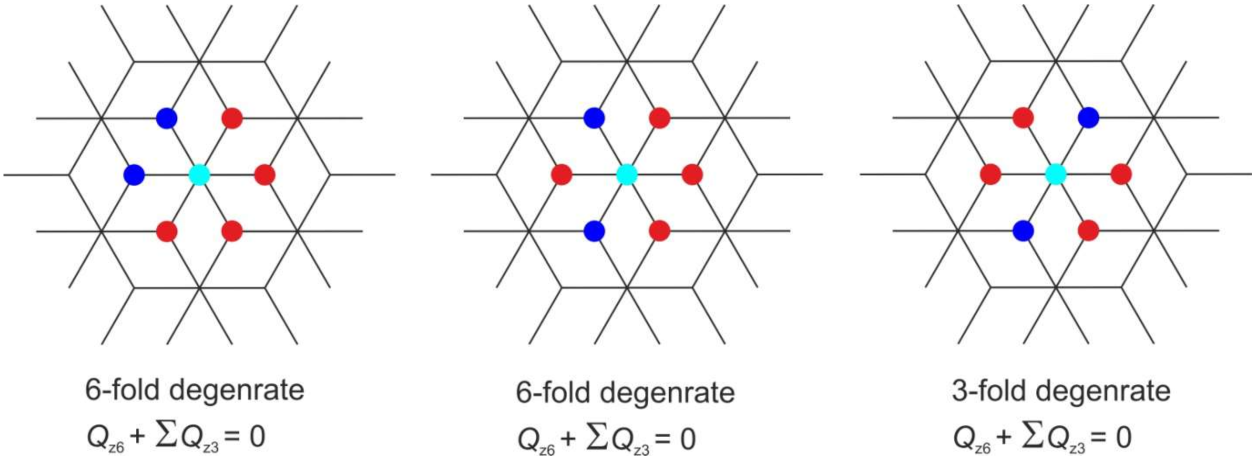

Supplementary Figure 6. Screening of emergent magnetic charge defects. From $2^6 = 64$ possible arrangements of $+q$ (blue circles) and $-q$ (red circles) emergent magnetic charges around a $+2q$ charge defect (cyan blue circle) there are only 6 + 6 + 3 = 15 charge configurations that result in an overall $Q_{z6} + \sum_{i=1}^{i=6} Q_{z3i} = 0$ magnetic charge, thus forming a screened state. Purely based on geometry, the probability to observe a screened state is 15/64 ≈ 0.23. The same applies to screening of a -2q charge.

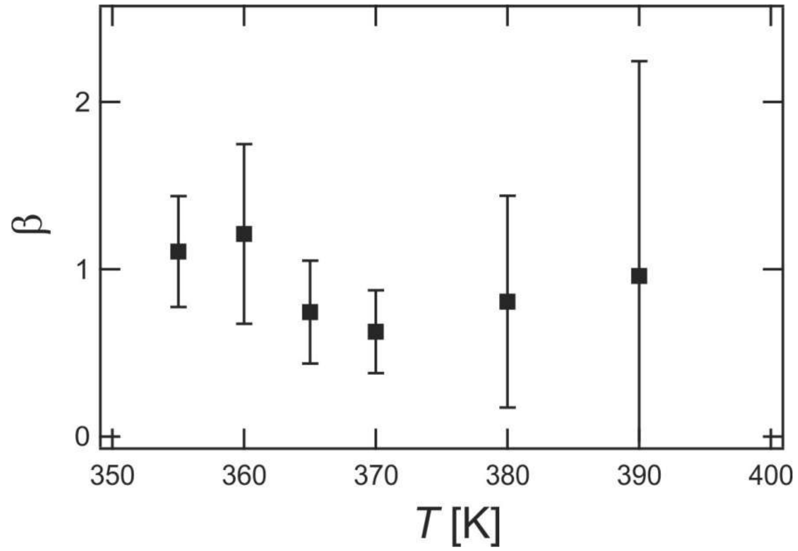

Supplementary Figure 7. Temperature dependence of the stretch parameter. The stretch parameter β is plotted as a function of temperature, as derived from thermal relaxation curves of screened magnetic charge defects. No clear dependence on temperature is found. Error bars are estimated by using the bootstrap method (see methods in the main text).

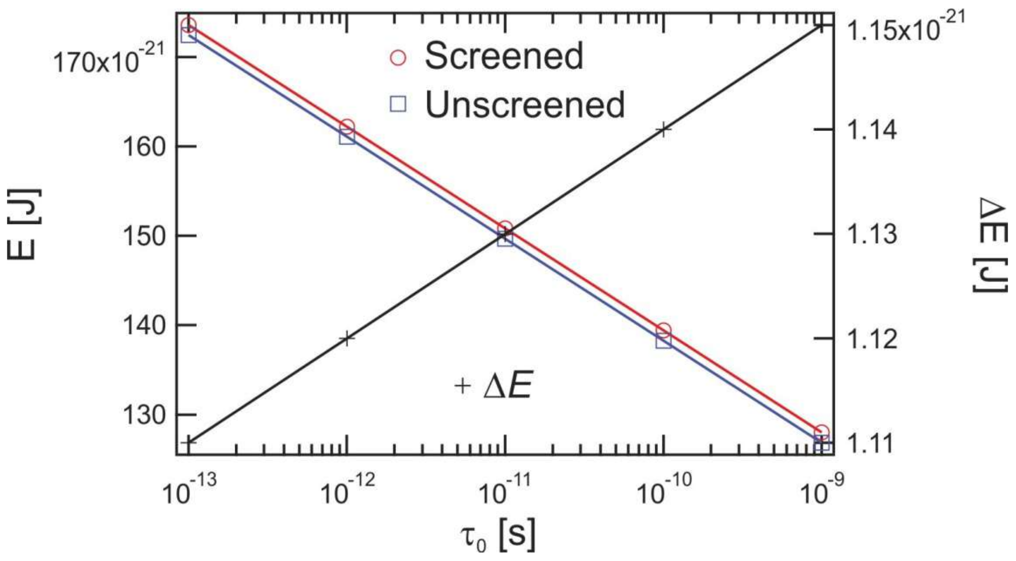

Supplementary Figure 8. Activation energies of screened- and unscreened charge defects. Derived activation energy of screened (red circles) and unscreened (blue squares) charge defects for different attempt time τ_0. While the absolute value of the activation energy varies with attempt time, the difference in activation energy (ΔE) remains relatively unaffected (black crosses).
